# The Discovery of Stromatolites Developing at 3570 m above Sea Level in a High-Altitude Volcanic Lake Socompa, Argentinean Andes

**DOI:** 10.1371/journal.pone.0053497

**Published:** 2013-01-07

**Authors:** María E. Farías, Nicolás Rascovan, Diego M. Toneatti, Virginia H. Albarracín, María R. Flores, Daniel G. Poiré, Mónica M. Collavino, O. Mario Aguilar, Martin P. Vazquez, Lubos Polerecky

**Affiliations:** 1 Laboratorio de Investigaciones Microbiológicas de Lagunas Andinas (LIMLA), Planta Piloto de Procesos Industriales Microbiológicos (PROIMI), CCT, CONICET, San Miguel de Tucumán, Tucumán, Argentina; 2 Instituto de Agrobiotecnologia Rosario (INDEAR), Rosario, Santa Fe, Argentina; 3 Facultad de Ciencias Naturales e Instituto Miguel Lillo, Universidad Nacional de Tucumán, San Miguel de Tucumán, Tucumán, Argentina; 4 Max-Planck Institute for Chemical Energy Conversion, Mülheim an der Ruhr, Germany; 5 Centro de Investigaciones Geológicas, Universidad Nacional de La Plata-CONICET, La Plata, Argentina; 6 Instituto de Biotecnología y Biología Molecular (IBBM), Universidad Nacional de La Plata-CONICET, La Plata, Argentina; 7 Max-Planck Institute for Marine Microbiology, Bremen, Germany; National University of Singapore, Singapore

## Abstract

We describe stromatolites forming at an altitude of 3570 m at the shore of a volcanic lake Socompa, Argentinean Andes. The water at the site of stromatolites formation is alkaline, hypersaline, rich in inorganic nutrients, very rich in arsenic, and warm (20–24°C) due to a hydrothermal input. The stromatolites do not lithify, but form broad, rounded and low-domed bioherms dominated by diatom frustules and aragonite micro-crystals agglutinated by extracellular substances. In comparison to other modern stromatolites, they harbour an atypical microbial community characterized by highly abundant representatives of *Deinococcus-Thermus*, *Rhodobacteraceae*, *Desulfobacterales* and *Spirochaetes*. Additionally, a high proportion of the sequences that could not be classified at phylum level showed less than 80% identity to the best hit in the NCBI database, suggesting the presence of novel distant lineages. The primary production in the stromatolites is generally high and likely dominated by *Microcoleus* sp. Through negative phototaxis, the location of these cyanobacteria in the stromatolites is controlled by UV light, which greatly influences their photosynthetic activity. Diatoms, dominated by *Amphora* sp., are abundant in the anoxic, sulfidic and essentially dark parts of the stromatolites. Although their origin in the stromatolites is unclear, they are possibly an important source of anaerobically degraded organic matter that induces *in situ* aragonite precipitation. To the best of our knowledge, this is so far the highest altitude with documented actively forming stromatolites. Their generally rich, diverse and to a large extent novel microbial community likely harbours valuable genetic and proteomic reserves, and thus deserves active protection. Furthermore, since the stromatolites flourish in an environment characterized by a multitude of extremes, including high exposure to UV radiation, they can be an excellent model system for studying microbial adaptations under conditions that, at least in part, resemble those during the early phase of life evolution on Earth.

## Introduction

Microbialites are organo-sedimentary deposits accreted by sediment trapping, binding and *in situ* precipitation due to the growth and metabolic activities of microorganisms [1], [2]. Geological records indicate that microbialites first appeared 3.5 Ga ago and were the main evidence of life on Earth for the next 2 Ga [3], [4]. Stromatolites are layered forms of microbialites. As the first communities performing significant oxygenic photosynthesis, they are thought to have played a major role in oxygenation of the Earth's atmosphere [5], [6]. The dramatic decline in the abundance and diversity of stromatolites, which occurred from 1 to 0.7 Ga ago, has been linked to the evolution and diversification of grazing, burrowing, and possibly boring metazoans [7]–[9].

Presently, actively forming stromatolites are found in habitats with diverse environmental conditions, ranging from extreme to moderate. Examples of well studied habitats include the hypersaline region of Hamelin Pool (HP), Western Australia [10], [11], hot springs such as Obsidian Pool (OP) in Yellowstone National Park [12], Shionoha (SHS), Japan [13], [14], or Frying Pan Lake (FPL), New Zealand [15], open marine waters of Exuma Sound (ES), Bahamas, [16], [17], or freshwater bodies at the Cuatro Ciénegas Basin (CCB), Mexico [18], [19], or Ruidera Pools (RP), Spain [20]. With the exception of OP, which lies about 2400 m above sea level (masl), a common characteristic of these habitats is their low to medium altitude (HP and ES: 0 m; SHS: 340 m; FPL: 460 m; RP and CC: around 800 m).

In this study we report on the discovery of stromatolites forming at the shore of a high-altitude volcanic lake Socompa (3570 masl). To the best of our knowledge, this is so far the highest altitude where actively forming stromatolites have been found. Following the preliminary characterization by Farías and colleagues [21], this study provides a more detailed description of the stromatolites with respect to the characteristics of their habitat, composition of their mineral phase and microbiota, and some aspects of microbial activity and physiology. We compare the Socompa stromatolites with other modern stromatolites, and hypothesize how the combined effects of the environment and the *in situ* microbial activity determine the stromatolites formation.

## Results

### General Physico-chemical Setting

**Figure 1:**
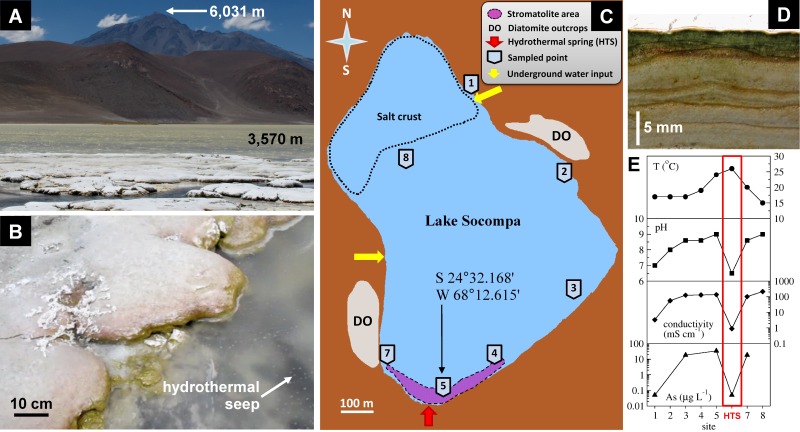
Socompa stromatolites and their habitat. (A) Stromatolite site at the lake shore. (B) Typical stromatolite bioherm. (C) Schematic diagram of the Socompa lake, showing notable sites in and around the lake (see legend). (D) Vertical section of the stromatolite. (E) Distribution of temperature, pH, conductivity and arsenic in the waters sampled along the lake shore in sampling points marked in panel C.

The lake Socompa is located in the high-altitude Andean plateau region known as the Puna, far away from any significant urban population. It is placed at the base of the still active volcano Socompa, in a basin surrounded by fossil diatomite outcrops (Figure 1). At the Alto Chorillo weather station, which is located at an altitude of 4800 m and about 180 km from the Socompa lake, the mean monthly air temperatures vary between 3°C in December-February (summer) and −4°C in July-August (winter), diurnal fluctuations reach about 11°C, and maximal global solar irradiances reach 1400 W m^−2^ in summer and 800 W m^−2^ in winter (Federico Bareilles, personal communication).

The stromatolites are found along the southern shore of the lake, in an area where a small stream and a number of seeps bring hydrothermal water (26°C) from a modern Andean volcanic system [22] into the lake (Figure 1). The site is exposed to air from about December to May, and submersed under 0.5–1 m of water for the rest of the year. During the February field campaign, the lake water at the stromatolites site was relatively warm (20–24°C), alkaline, rich in dissolved ions such as Na^+^, K^+^, Mg^2+^, Ca^2+^, Cl^−^ and SO_4_^2−^ (total conductivity up to 130 mS cm^−1^), rich in organic carbon, nitrate, phosphate, silicate and iron, and contained a strikingly high amount of arsenic (Table 1). In contrast, the hydrothermal stream water was slightly acidic, had about 100-fold lower conductivity, 10-fold lower concentrations of nitrate and phosphate, and by about 10% higher concentrations of silicate (Table 1). Phytoplankton in the lake water was dominated by diatoms and cyanobacteria, but the count of viable cells was low (∼200 cells ml^−1^), consistent with the very low content of detected chlorophyll *a* (Chl *a*; 3 µg l^−1^). Except for the sites with the hydrothermal and non-hydrothermal water input, the water along the lake shore was consistently alkaline, hyper-saline and rich in arsenic (Figure 1e).

**Table 1:** Basic physico-chemical and biological characteristics of the water samples from the Socompa lake (site 5 in Figure 1) and from the hydrothermal spring (HTS).

		Site 5	HTS
Temperature	(°C)	20–24	26
pH		8.6	6.5
Total hardness	(mg CaCO_3_ l^−1^)	22,788	231
Conductivity	(mS cm^−1^)	115	0.9
Sodium	(mg l^−1^)	37,113	91
Potassium	(mg l^−1^)	5,298	12.8
Magnesium	(mg l^−1^)	4,090	20.9
Calcium	(mg l^−1^)	2,383	59.5
Chloride	(mg l^−1^)	52,234	255
Sulfate	(mg l^−1^)	31,847	322
Arsenic	(mg l^−1^)	18.5	0.05
Nitrate	(mg l^−1^)	59	6.5
Phosphate	(mg l^−1^)	25	2.5
Silicate	(mg l^−1^)	73	82.6
Iron	(mg l^−1^)	1	<0.1
Manganese	(mg l^−1^)	<0.05	0.5
Total organic carbon	(mg l^−1^)	50	1.9
Cell count	(cells ml^−1^)	200	NA
Chlorophyll *a*	(µg l^−1^)	3	NA

### Basic Description of the Stromatolites

Stromatolites form broad, rounded and low-domed bioherms (up to 24×80 cm in size). Neighbouring domes tend to coalesce into bigger domed biostromes (Figure 1a–b). Vertical sections display visually clear stratification (Figure 1d). The surface continuously exposed to air is covered by a white-pinkish crust, whereas the surface intermittently exposed to water due to waves is green-yellow. Below this cover is a 0.5–1.5 mm thick dark-green layer, which gradually fades away and disappears at depths of 3–5 mm. Deeper parts are characterized by alternating light-brown (5–20 mm thick) and dark-brown (0.5–1.5 mm thick) layers. Black spots are occasionally found in the darker layers, presumably a sign of a volcanic ash deposition.

**Figure 2:**
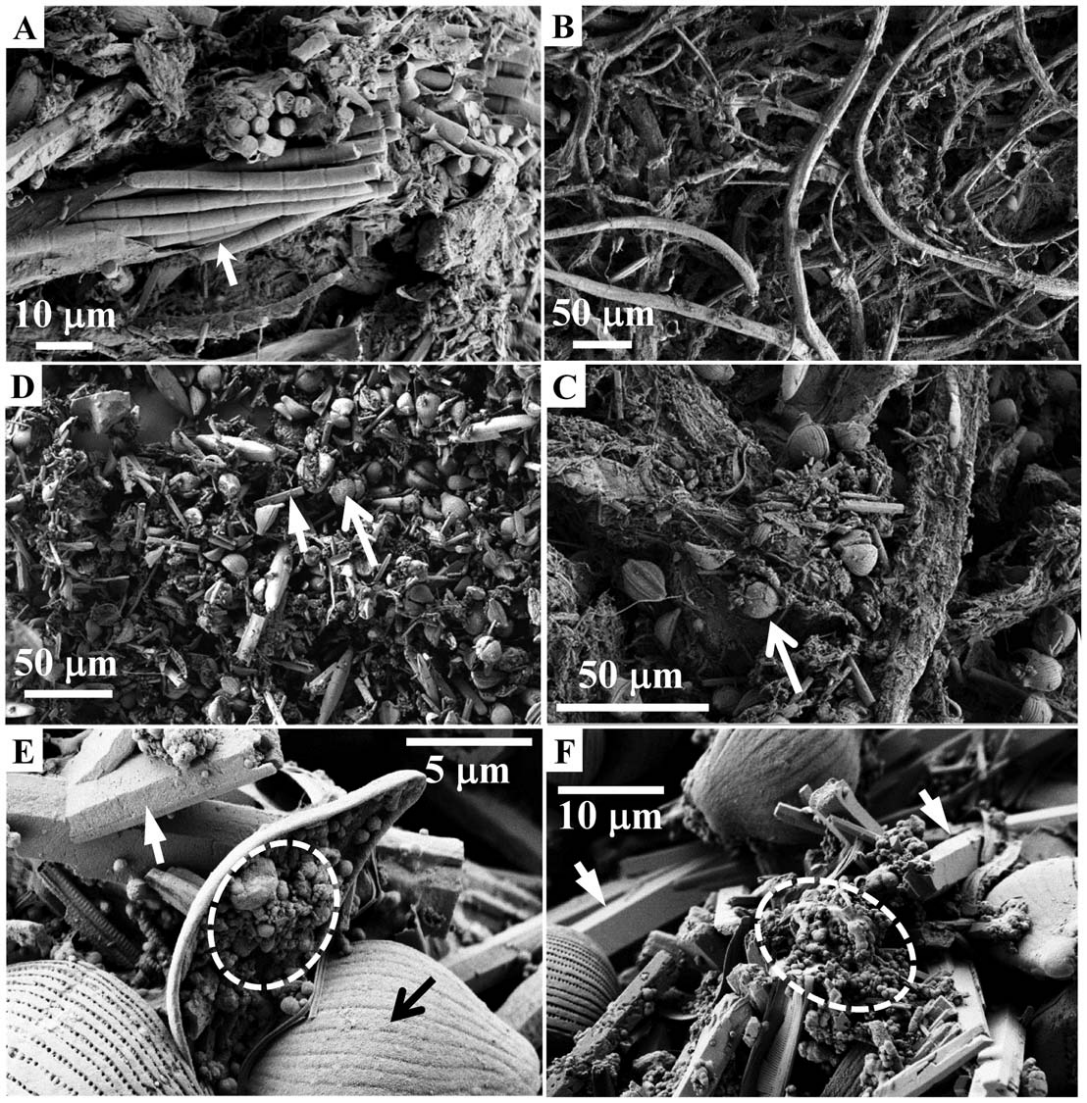
Electron micrographs of the Socompa stromatolite samples. Arrows point to the dominant morphotypes of cyanobacteria (*Microcoleus* sp., panel A) and diatoms (*Amphora* sp., panel C–E), and to the dominant minerals (aragonite, panels D–F). Dashed circles in panels E–F mark locations with abundant colonization by prokaryotic cells, as derived based on their size and morphology.

Light and electron microscopy revealed that filamentous cyanobacteria dominated the top ∼2 mm of the stromatolites (Figure 2a–b). Diatoms and mineral micro-crystals were also present in relatively high amounts, and appeared agglutinated to the cyanobacterial filaments and sheaths (Figure 2c). A significant proportion of the diatom frustules, however, lacked visible chloroplasts. Deeper stromatolite layers were dominated by diatom frustules and mineral micro-crystals with diameters of 1–4 µm and lengths up to 30 µm (Figure 2d). The proportion of disrupted or crushed diatom frustules, as well as the abundance of micro-crystals, appeared to increase with depth. Colonization by prokaryotic organisms was also highly apparent (Figure 2e–f), especially in deeper layers. Microscopical observations of many stromatolite samples combined with identification based on morphology (data not shown) revealed that the cyanobacterial genera included *Microcoleus* (dominant), *Anabaena, Aulosira, Lyngbya, Oscillatoria* and *Myxosarcina*, while the diatom genera included *Amphora* (dominant), *Navicula, Pinnularia, Eucocconeis, Cymbella* and *Synedra*. At depths >5 mm, the mineral microcrystals were on average larger and more abundant in the visually brighter layers than in the darker layers. Furthermore, the darker layers contained abundant remnants of bundles of *Microcoleus* filaments in variable stages of decomposition (orange in colour), whereas these were largely absent in the brighter layers.

The bulk stromatolite (top 5 cm) contained a relatively high amount of water (porosity 50%) and organic matter (18% of dry weight). The ICP-AES and ICP-MS analyses of the oxidized mineral solid phase revealed the dominance of SiO_2_ (33.8%), consistent with the high abundance of diatom frustules. Other components included CaO (17.95%), Na_2_O (5.67%), MgO (4.45%), Al_2_O_3_ (1.65%), K_2_O (1.07%), Fe_2_O_3_ (0.6%), P_2_O_5_ (0.07%) and MnO (0.01%), suggesting the presence of minerals such as calcium carbonates, feldspar, halite, and possibly of clay minerals. The presence of these minerals was confirmed by X-ray diffraction, which further showed that aragonite dominated over calcite (see Ref. [21]).

### Microenvironmental Conditions

**Figure 3:**
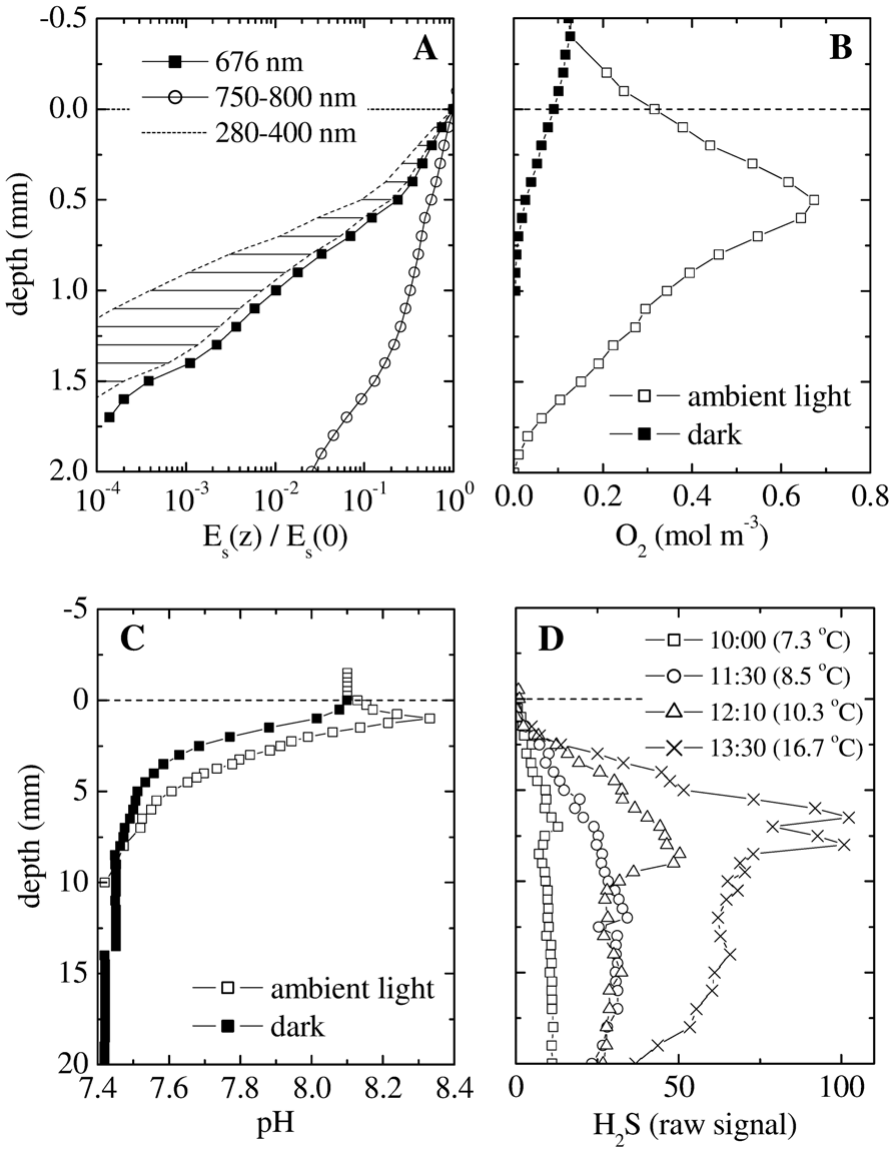
Typical profiles of scalar irradiance (E_s_), O_2_, pH and H_2_S in the Socompa stromatolites, as measured with microsensors in the field at 3500 masl. (A) E_s_ was normalized to the value at the stromatolite surface, and is shown for selected wavelengths (see legend). E_s_ in the UV region was estimated based on the profiles measured at 676 nm and 750 nm. (B–C) Steady state profiles of O_2_ and pH were measured at 25°C. Incident intensity of the ambient PAR was 130 W m^−2^ (corresponding to 600 µmol photons m^−2^ s^−1^). (D) Transient profiles of H_2_S were measured in a shaded place (PAR intensity <50 µmol photons m^−2^ s^−1^) during gradually increasing ambient temperature (see legend). For all measurements salinity of the overlying water was 48 g L^−1^, as estimated from the measured conductivity (70 mS cm^−1^) assuming seawater salt composition. Due to the lack of calibration standards, H_2_S is given only as a raw signal linearly proportional to H_2_S concentrations. Depth 0 corresponds to the stromatolite surface. Note the 10-fold difference in the depth scale in panels A–B and C–D.

Microsensor measurements revealed steep gradients of scalar irradiance, O_2_, pH and H_2_S in the top few millimeters of the stromatolite (Figure 3). The gradient of scalar irradiance was highly wavelength-dependent (Figure 3a). For example, the decrease across the top 1 mm was 3-fold in the near infrared region (750–800 nm) and 100-fold at the wavelength of maximal *in vivo* absorption by Chl *a* (676 nm). The decrease in the UV region (280–400 nm), estimated as described in Supplement S1, was even more pronounced, ranging from 140-fold to 2500-fold across the top 1 mm. This pronounced attenuation of UV light was due to the combined effects of intense scattering and absorption (see Figure S2 in Supplement S1).

Under constant ambient illumination, O_2_ reached a pronounced maximum of about 650 µM (corresponding to ∼510% of air saturation at ambient pressure) at depth of 0.5 mm, and penetrated to a maximal depth of 2 mm (Figure 3b). In contrast, maximal O_2_ penetration in the dark was only about 0.8 mm. Porewater pH depended significantly on illumination in the top 5 mm (Figure 3c), decreasing steeply with depth in the dark and showing a modest peak of ∼8.3 at 0.5 mm depth at ambient light. Deeper in the stromatolite, pH reached about 7.4 (∼0.7 pH units lower than in the overlying water) and was light independent. H_2_S was detectable at depths from about 2 mm until at least 20 mm (Figure 3d). Increasing temperature resulted in a gradual increase in H_2_S concentrations, which was most pronounced at depths from about 5 to 20 mm. This suggested that sulphate reduction, the most likely process responsible for H_2_S production in the stromatolites, was most active in this zone and was likely stimulated by temperature.

### Pigments

**Figure 4:**
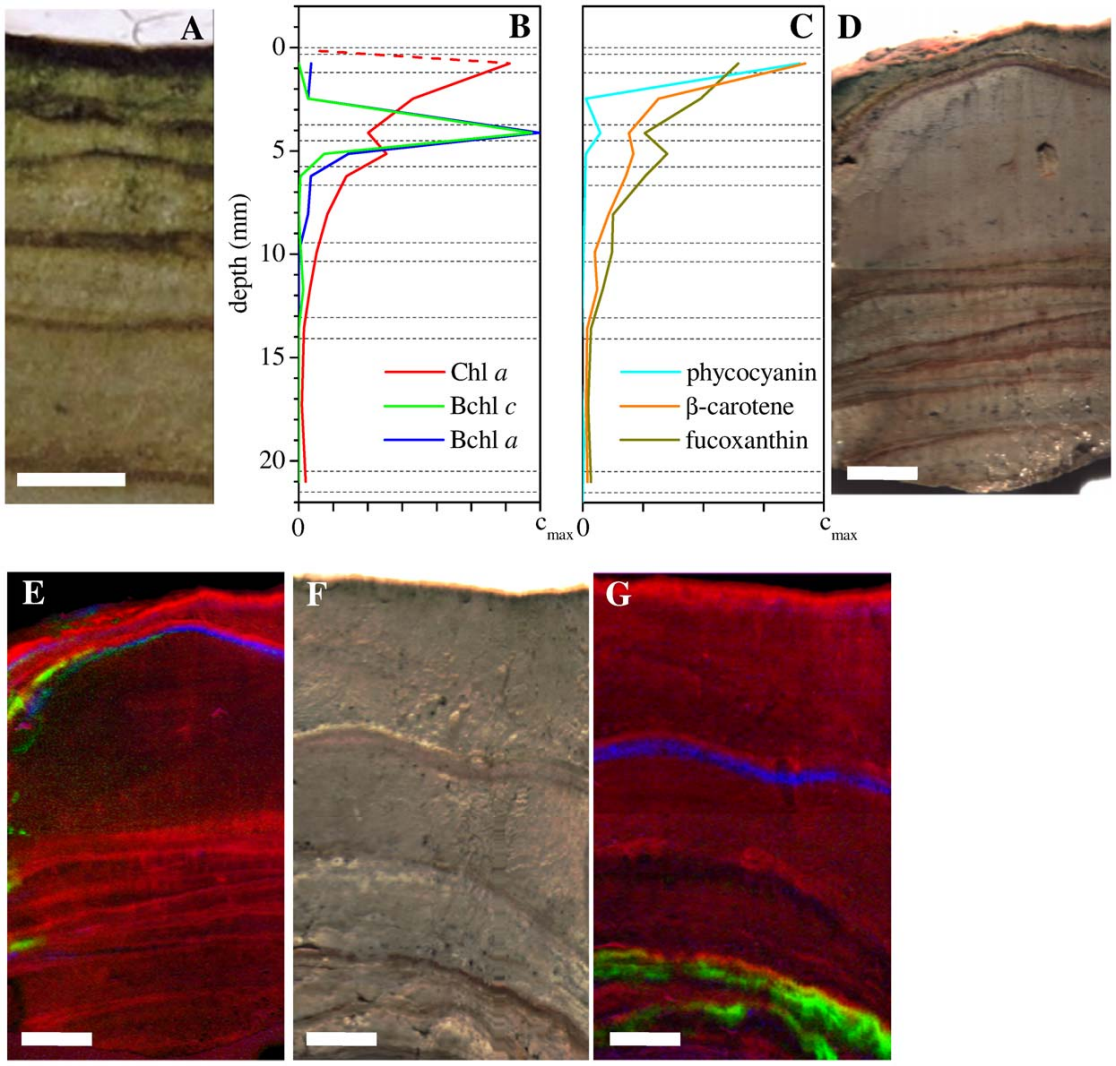
Distributions of pigments in the Socompa stromatolites. (A) Vertical section of the stromatolite sample measured by HPLC, with the corresponding pigment profiles shown in panels B–C. Dashed horizontal lines indicate boundaries of the sampled layers. The scaling of the concentration axis differs between pigments: c_max_ = 70, 2.1, 7 and 400 µg (g dw) ^−1^ for Chl *a*, Bchl *a*, Bchl *c* and phycocyanin, respectively. β-carotene and focoxanthin are given in relative units only. Note that pigment concentrations are not shown in the top layer (0–0.33 mm) because they could not be measured by HPLC due to an insufficient amount of sample. In this layer, the Chl *a* concentration was estimated based on the profile derived from hyper-spectral imaging as 10% of the maximum (dashed line). (D–G) True-color and the corresponding false-color pigment images, obtained by hyper-spectral imaging of two additional stromatolite samples. Intensity of the red, green and blue colors in images E and G is proportional to the concentration of Chl *a*, Bchl *c* and Bchl *a*, respectively. Scale bar = 5 mm in all images.

HPLC analysis revealed that Chl *a* reached a maximum of 61 µg (g dw) ^−1 ^in the sub-surface dark-green layer (depth interval 0.3–1.2 mm), and decreased approximately exponentially at depths >1 mm. In contrast, bacteriochlorophylls (Bchl) *a* and *c* co-occurred in a distinct layer at depths from 4 to 5 mm (Figure 4a–b). Hyperspectral imaging showed, however, that this pattern was not general (Figure 4d–g). For example, Chl *a* concentrations often locally increased several centimetres below the stromatolite surface, in distinct bands that coincided with the visually darker layers (this pattern was confirmed by HPLC). Furthermore, the depths of the Bchl *a* and Bchl *c* containing layers varied considerably amongst stromatolite samples as well as within the same sample, ranging from 4 mm up to 4 cm. The layers containing Bchl's *a* and *c* were generally slightly below (but overlapping) the layers with the locally increased Chl *a* content. The Bchl *c* containing layers were usually deeper than those containing Bchl *a*, but sometimes they were absent or their relative position was even reversed (Figure 4d–g).

The cyanobacteria-specific accessory pigment phycocyanin was detected mostly in the dark-green subsurface layer, whereas the profile of the diatom-specific accessory pigment fucoxanthin correlated (R = 0.986, p<2×10^−4^) with that of Chl *a* at depths >1.2 mm (Figure 4b–c). This shows that Chl *a* in deeper stromatolite layers is due to diatoms and not due to cyanobacteria, and suggests that viable diatom cells are present in significant amounts in anoxic and sulfidic parts of the stromatolites (compare Figures 3b–c and 4b–c).

### Bacterial Diversity Based on 16S Pyrotags

Clustering of the 113,255 bacterial 16S pyrotags at similarity levels of 0.97, 0.90 and 0.80 resulted in 2776, 1147 and 293 Operational Taxonomic Units (OTUs), respectively. These values were reduced to 1352, 683 and 216, respectively, when OTUs with ≤2 reads (singletons and doubletons) were eliminated. None of the OTU estimates reached an asymptote in a rarefaction curve at 0.97 (data not shown), suggesting that the actual number of OTUs in the sample was even higher than estimated by the present sampling effort.

**Figure 5:**
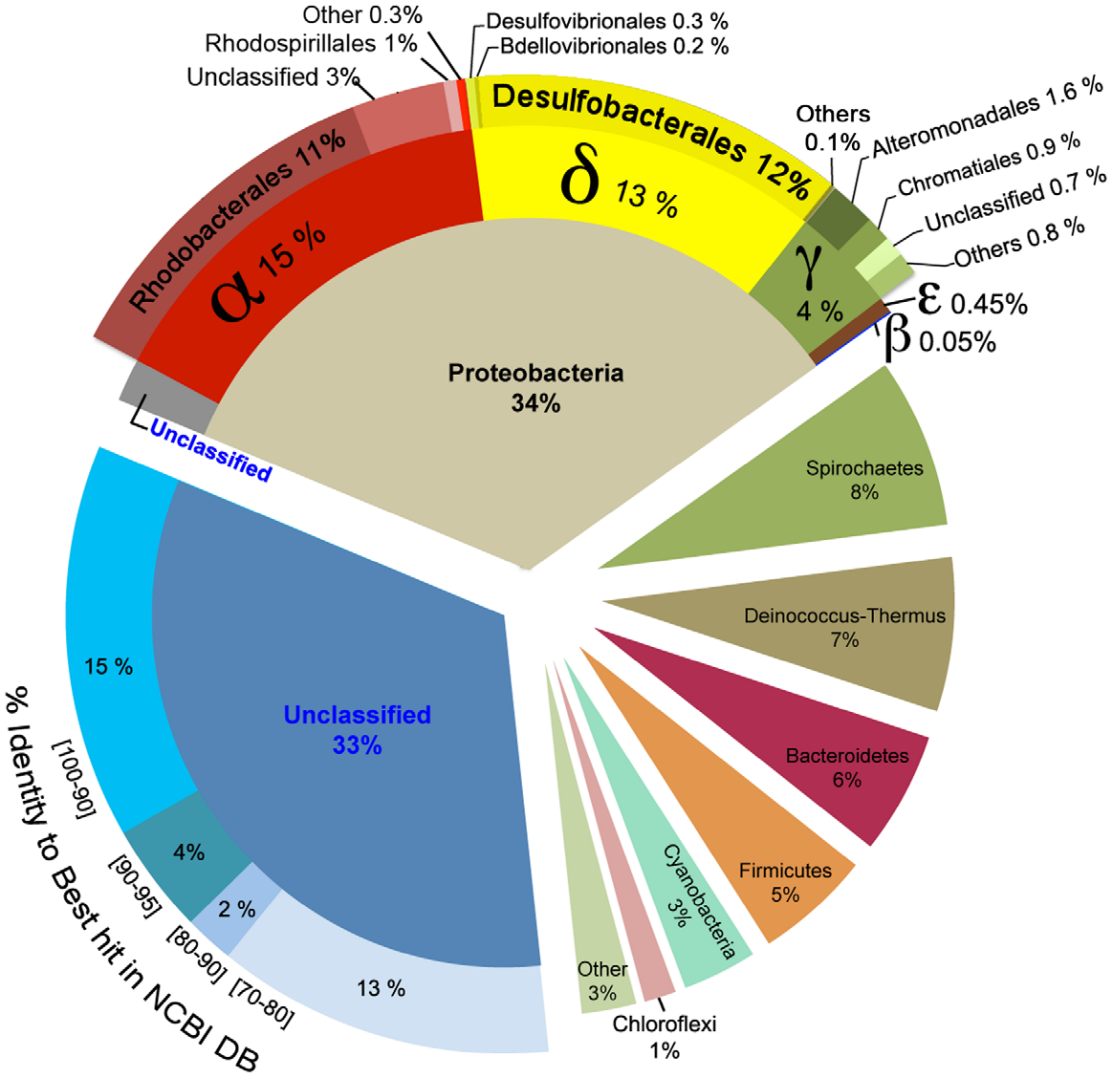
Taxonomic composition of *Bacteria* in the Socompa stromatolites derived from the detected 16S pyrotags. The unclassified sequences at phylum level were analysed by BLAST against the full NCBI database, and grouped according to the percentage identity to the best hit.

About one third of the OTU’s at 0.97 similarity could not be classified at phylum level (Figure 5). Furthermore, 13% of all sequences showed <80% similarity to the best hit in the full NCBI database (including environmental sequences) by Blast, suggesting that they could belong to unknown distant lineages. This was further supported by a tree constructed from all aligned OTU representative sequences, which showed that these abundant unclassified sequences clustered into two different and well-defined groups that strongly separated from all other OTUs (data not shown).

Among the classified OTUs (at 0.90 similarity), *Protebacteria* represented the most abundant phylum (34% of all sequences; Figure 5), with majority of sequences related to *Alpha-* (15%), *Delta-* (13%) and *Gammaproteobacteria* (4%), and very few *Epsilon-* (0.45%) and *Betaproteobacterial* (0.05%) sequences. *Delta-proteobacteria* were dominated by *Desulfobacterales*, whereas *Alpha-proteobacteria* were dominated by *Rhodobacterales* and an unclassified group. Other abundant phyla, representing 33% of all sequences, included *Spirochaetes* (8%), *Deinococcus-Thermus* (7%), *Bacteroidetes* (6%), *Firmicutes* (5%), *Cyanobacteria* (3%, dominated by the genus *Microcoleus*) and *Chloroflexi* (1%). Sequences corresponding to the 16S rRNA gene of chloroplasts from the *Bacillaryophyta* (diatoms) represented 1% of all sequences (data not shown).

### Diversity of the *nif*H and *amo*A genes

Numbers of OTUs (obtained at 0.95 similarity) identified in the *nif*H and *amo*A gene libraries were close to those predicted by the CHAO1 richness estimator, suggesting that the obtained libraries represented well the diversity of diazotrophic and nitrifying bacteria in the stromatolites. These results were supported by rarefaction curves (data not shown).

Taxonomic assignments of the analyzed *nif*H sequences suggested that nitrogen fixation in the stromatolites is potentially performed by highly diverse organisms, including *Cyanobacteria, Beta*-, *Gamma*- and *Deltaproteobacteria* (Table 2). Three out of 39 *nif*H sequences showed high similarity to those of *Microcoleus chthonoplastes* (97%), whereas 22 *nif*H sequences clustered with those of the sulphate reducing bacteria *Desulfovibrio* sp. and *Desulfatibacillum* sp. The identity of the latter sequences was, however, rather low (81–82%). A relatively large portion (7 out of 39) of the *nif*H sequences showed as little as 70–75% identity to the closest cultured relative and 80–87% to any other nitrogenase sequences in the GenBank (Table 2), suggesting that a significant part of the diazotrophic community may be novel.

**Table 2:** Relatedness of the *nif*H and *amo*A gene sequences detected in the Socompa stromatolites to those in the most closely related cultivated isolates and to the closest hits in the NCBI database.

				Nearest cultivated				Nearest database sequence						
Gene	Group	N	Phylogenetic affiliation	name	accession number	% identity		clone name		accession number	% identity			habitat
*nif*H	Enif1	22	δ-proteobacteria	*Desulfatibacillum alkenivorans*	CP001322	81–82		GN823A25		AY244738	94–95			saline mats
	Enif2	6	Spirochaetes	*Spirochaeta smaragdinae* DSM 11293	CP002116	70–71		MO175A12		AY221803	86–87			Mono Lake
	Enif3	1	Chlorobiales	*Chlorobium phaeobacteroides* BS1	CP001101	75		07-II.11		GU193538	80			saline mats
	Enif4	3	Cyanobacteria	*Microcoleus chthonoplastes*	GQ397260	97		same as nearest cultivated						
	Enif5	3	γ-proteobactereia	*Ectothiorhodospira mobilis*	EF199954	86–87		08 II.118		GU193882		90–91	saline mats	
	Enif6	3	γ-proteobactereia	*Halorhodospira halophila*	AB189641	87		same as nearest cultivated						
	Enif7	1	β-proteobactereia	*Burkholderia vietnamiensis*	AM110707	98		JUL_H04	EF568503		99		oligotrophic ocean	
*Amo*A	Eamo1	11	β-proteobactereia	*Nitrosomonas sp.* ML1	AY958703	83		17–11	EU116356		97–100		Salar de Huasco	
	Eamo2	9	β-proteobactereia	*Nitrosomonas* sp. IWT310	DQ228467	83		10–27	EU116360		97–98		Salar de Huasco	
	Eamo3	1	β-proteobactereia	*Nitrosospira* sp. En13	EF175097	85		–	AB360859		90		soil	

The use of *amo*A primers specific for ammonia-oxidizing archaea (AOA) and *Gammaproteobacteria* yielded no PCR amplification product, whereas the *Betaproteobacterial amo*A genes were successfully amplified using the amoA-1F-amoA-2R primers. Most clones (20 out of 21) grouped with the *Nitrosomonas* lineage, one with *Nitrosospira*, in both cases with a relatively low identity (83–85%) to cultured bacteria and, for the *Nitrosomonas*, with a high identity (97–100%) to sequences from other hyper-saline environments (Table 2).

### Effect of UV Irradiation

**Figure 6:**
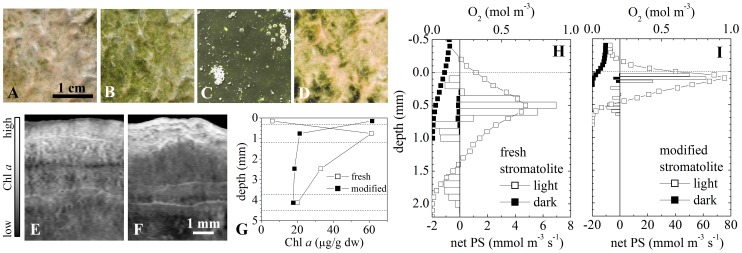
Effect of UV irradiation on the distribution and activity of cyanobateria in the stromatolites. (A–D) Appearance of the stromatolite surface under various light conditions: (A) full ambient light at 3500 masl, (B) after 1–2 hours of incubation in a shade at 3500 masl, (C) after 5 days of incubation at ambient light conditions with the UV light intensity reduced by about 30% (at 460 masl), (D) after 2–3 hours of exposure to artificial UV-B radiation at 460 masl. Note that because of the extremely dark-green appearance of the surface in panel C (reflectance <1%), the brightness of the shown image was increased 5-fold. (E–G) Vertical distributions of Chl *a* in the stromatolite obtained by hyperspectral imaging and HPLC. Panels E and F correspond to samples shown in panels A and B, while open and closed symbols in panel G correspond to samples shown in panels A and C, respectively. (H–I) Steady state profiles of oxygen and of the volumetric rates of net photosynthesis (net PS) in the freshly collected (shown in A) and modified (shown in C) stromatolite. Shown are profiles measured at incident PAR intensities of 600 µmol photons m^−2^ s^−1^ (open symbols and bars) and in the dark (filled symbols and bars). Note the 10-fold difference in the scaling of the net PS axis.

The appearance of the stromatolite surface changed from white-pinkish to light-green when the sample was kept in a shade at 3500 masl (Figure 6a–b). This change occurred within 1–2 hours, and was fully reversible when the sample was re-exposed to the full ambient light at 3500 masl. When the sample was subsequently incubated for 5 days at ambient light conditions at an altitude of 430 masl, which were similar except for the reduced intensity in the UV-B region (by an estimated 30%; [23]), the surface colour of the stromatolite changed to dark-green (Figure 6c). After this modification, irradiation of the sample with an artificial UV-B light (intensity 0.8 W m^−2^) resulted again in a change of the surface colour from green to white-pinkish (Figure 6d), which was also reversible when the UV-B exposure was removed. Microscopy and Chl *a* analysis by hyper-spectral imaging and HPLC revealed that both the short- and long-term reversible colour changes were linked to the vertical shifts in the distribution of the dominant cyanobacteria (*Microcoleus* sp.) in the stromatolite: while the population was concentrated in a sub-surface layer (in a depth interval 0.3–1.2 mm) in the sample exposed to full ambient light at 3500 masl, it became concentrated in the top 0.3 mm in the shade-incubated sample or in the sample incubated at full ambient light at 430 masl (Figure 6e–g). Together, this shows that the distribution of the dominant cyanobacteria in the stromatolites is strongly controlled by ambient illumination, most likely through negative phototaxis towards UV-B.

Clearly, as implied by the light microsensor measurements, migration of the *Microcoleus* sp. filaments deeper into the stromatolite not only helps them lower the exposure to a potentially harmful light (UV-B), but also results in a lower exposure to the light that they require for photosynthesis (photosynthetic active radiation, PAR). To estimate the effects of a short-term (days) reduction in the UV intensity of the ambient light on the activity in the oxic zone of the stromatolite, we compared the rates of photosynthesis and respiration in the freshly collected stromatolite with those in the stromatolite modified by the 5 day incubation at 430 masl (Figure 6h–i). At the incident intensity of 600 µmol photons m^−2^ s^−1^ in the PAR region, the layer with positive net photosynthesis in the fresh stromatolite extended from 0.25 to 0.75 mm, and the total flux of oxygen exported from this layer was about 1.9 µmol m^−2^ s^−1^, with 45% diffusing upwards to the overlying water and 55% downwards. In contrast, the net photosynthetic production in the modified stromatolite was concentrated in the top 200 µm, and the total exported oxygen flux was about 4.5-fold larger (8.6 µmol m^−2^ s^−1^), with 70% diffusing upwards and 30% downwards. The volumetric rates of net photosynthesis increased about 10-fold, from 4–7 mmol m^−3^ s^−1^ to astonishing 50–60 mmol m^−3^ s^−1^ (compare open bars with net PS>0 in Figure 6h and 6i). Respiration rates close to the oxic-anoxic interface also dramatically differed, increasing from around 0.8–1 mmol m^−3^ s^−1^ in the fresh stromatolite to 7–9 mmol m^−3^ s^−1^ in the modified stromatolite. A similar stimulation of respiration was observed also in the dark, with fluxes decreasing from −0.14 to −0.4 µmol m^−2^ s^−1^ and the volumetric rates increasing from 0.25 to 3 mmol m^−3^ s^−1^ in the fresh compared with the modified stromatolite, respectively (filled bars in Figure 6h and 6i).

## Discussion

### Mineral Composition of the Stromatolites

In comparison to other modern stromatolites, the Socompa stromatolites do not appear to lithify, and although they do contain a high abundance of CaCO_3_ precipitates (mostly as aragonite micro-crystals), their mineral fraction is by far dominated by opal (amorphous SiO_2_.*n*H_2_O, either as whole or crushed diatom frustules).

Two critical aspects are recognized in the context of microbially induced CaCO_3_ precipitation in modern stromatolites and microbial mats: the “alkalinity engine,” i.e., a process that generates carbonate alkalinity to promote precipitation, and the presence of an organic exopolymeric matrix (EPS), which is important as a site of mineral nucleation and growth but also as a medium with a metal-binding potential that may inhibit precipitation [24]–[27]. Photosynthesis and anaerobic organic matter degradation by sulphate reduction (SR) promote carbonate precipitation, whereas aerobic respiration promotes dissolution [28]. The high abundance of CaCO_3_ micro-crystals in deeper parts of the stromatolites, where active SR is strongly implicated by the sulfide microsensor measurements and further supported by the high abundance of sequences closely related to *Desulfobacterales*, suggests that carbonate precipitation in the Socompa stromatolites is driven by SR. The presence of carbonate minerals in the EPS-rich cyanobacterial layer suggests that photosynthetically driven carbonate precipitation may also occur, although entrapment of micro-crystals from the overlying water by the EPS formed in this layer can be another possibility. However, considering that their abundance is much lower in this layer, it is likely that neither of these processes plays a significant role for the growth of the Socompa stromatolites. From the available data, it is unclear why the stromatolites do not lithify. Although CaCO_3_ precipitation does seem to occur, the process of cementation does not, possibly because the EPS that agglutinate the structure together contain special functional groups that inhibit this process [26], [27]. More detailed analyses, especially of the porewater chemistry and properties of the EPS, are required to resolve this issue.

The high abundance of diatoms in the Socompa stromatolites, whose frustules dominate its mineral phase, is rather intriguing. The HPLC analysis revealed that pigmented, and thus presumably viable, diatom cells are present in highly sulfidic parts of the stromatolites where light in the visible spectral range practically does not penetrate (down to about 15 mm). This is not uncommon by itself, as diatoms have been shown to survive for many decades in dark sulfidic marine sediments [29]–[31]. In fact, through their ability to accumulate nitrate, diatoms may play an important role in the nitrogen cycle [32], or by supplying organic matter to SR bacteria, dying diatoms may be an important driver of the “alkalinity engine” and thus contribute to the *in situ* precipitation of CaCO_3_ minerals in the stromatolites. What is unclear is their origin. Our results show that cyanobacteria, mostly the filamentous *Microcoleus* sp., were by far the most dominant phototrophs in the euphotic zone of the stromatolites, whereas diatoms were scarce. Therefore it is most likely that cyanobacteria dominate also the primary production in the stromatolites.

A possible scenario that could explain the observed distribution of diatoms (viable cells and frustules) would be through episodes of rapid diatom growth at the stromatolite surface and subsequent recolonization of the stromatolite surface by the migrating filamentous cyanobacteria. This explanation would be consistent with the observed stratification of the stromatolites, which shows an alternating succession of layers with abundant remnants of filament bundles (most likely of *Microcoleus* sp.) in various stages of decomposition and layers dominated by the benthic diatom genus *Amphora* (with the cyanobacterial filaments largely absent). The rather large and variable thickness of the diatom-dominated layers (from 2 up to 15 mm) suggests that the benthic diatom proliferation would occur in episodes of high but variable intensity and variable duration. On the one hand, the occurrence of these events would be favoured by the relatively high availability of nutrients in the lake water. On the other hand, stressors such as high levels of UV radiation and arsenic may suppress them, suggesting that their occurrence will be controlled by an interplay between these factors.

Another possibility would be a transport by wind from the diatomite outcrops found around the lake. However, a brief check of one of the diatomites revealed that the dominant diatoms (*Navicula* sp.) differed from those dominating the stromatolites (*Amphora* sp.). Furthermore, even if such transport occurred, it would involve only fossil diatom frustules, not live cells that are plentiful in the top 15 mm of the stromatolites. Clearly, more frequent and long-term observations of the biotic and abiotic components in the lake water, as well as a more thorough exploration of the Socompa diatomites, are required to elucidate the origin and function of the highly abundant diatoms in the stromatolites.

### The Role of the Hydrothermal Input

The Socompa lake is located in one of the harshest environments on Earth. Due to the rainshadow effect of the Andes, the region experiences some of the lowest rates of precipitation (<200 mm per annum; [33]). Furthermore, due to the high altitude and low latitude, it receives some of the highest levels of light, with the global and UV solar irradiance reaching, respectively, up to 1500 W m^−2^ and 68 W m^−2^ [34], and the daily erythemal UV dose varying from 2 kJ m^−2^ in July up to 10 kJ m^−2^ in January [35] (for comparison on a global scale, see Ref. [36]). As a consequence, surface soils exhibit very high diurnal temperatures fluctuations (up to 40–66°C) and extreme cooling rates at subzero temperatures (1.5–1.8°C h^−1^). Additionally, the soils have generally very low organic carbon and nitrogen content [38]. The combination of these factors makes soils from this environment inhabitable generally only by a very low diversity of organisms, mostly bacteria and archaea, but also some fungi and protists [37], [38], [40].

As documented in previous studies, geothermal input in the form of fumaroles or warm springs gives rise to a locally increased diversity and abundance of life in this hostile environment [39], [40]. Such input provides reduced substances that can be utilized as an energy source for microbial metabolism. However, what appears to be even more important for a sustainable life in this environmental setting is the more stable and, to some degree, elevated temperature maintained in the vicinity of the geothermal input.

Based on the available data in this study, it seems likely that the same factor is most critical also for the formation of the Socompa stromatolites. First, the stromatolites are not found in other sites around the lake that lack hydrothermal input. Second, as revealed by the water chemistry analysis, the lake water contains essentially all components, and at much higher concentrations (except for silicate), as the hydrothermal spring does, making it unlikely that the input of these chemicals is critical for the stromatolites formation. Third, although the hydrothermal and lake waters have substantially different pH, microsensor measurements suggest that pH in the stromatolites is controlled by the microbial activity (photosynthesis and aerobic respiration in the top 2 mm and SR below) and not by the pH of the overlying water. Fourth, as suggested by sulfide microsensor measurements, sulphate reduction, which is the most likely process responsible for the *in situ* mineral precipitation in the Socompa stromatolites, is stimulated by temperature. Taken together, this evidence suggests that the hydrothermal water input facilitates the development of the Socompa stromatolites mostly by providing elevated and more stable temperatures, which stimulate the activity of SR bacteria and thus lead to a positive net rate of mineral precipitation. Although not tested in this study, temperature increase and stabilization may also have a positive effect on the phototrophy-based primary production, which is a critical source of organic matter for the SR-based “alkalinity engine”. Additional experiments and long-term observations are required to test these hypotheses.

### The Role of Cyanobacteria and UV Light

Cyanobacteria are generally considered crucial for the development of stromatolites, mainly due to their role as the dominant provider of organic carbon (especially as exopolysaccharides; [25]), or due to their direct involvement in mineral precipitation [14], [41]. Our data show that cyanobacteria play a dominant role in primary production, and potentially an important role in nitrogen fixation, in the Socompa stromatolites too. However, based on the differences in the amount of aragonite micro-crystals in the surficial (cyanobacteria-dominated) and deeper (diatom-dominated) stromatolite layers (see above), we assume that they do not play a significant direct role in *in situ* carbonate precipitation in the Socompa stromatolites. Furthermore, unlike in other modern stromatolites, the dominant cyanobacteria in the Socompa stromatolites are *Microcoleus* sp. (for comparison, see Ref. [42]–[45]).

Microscopy and pigment analyses revealed that these cyanobacteria concentrate in about a 1 mm thin layer located about 0.3 mm below the stromatolite surface, under a “cover” of a white-pinkish crust. This appears to be their adaptation strategy to minimize the exposure to potentially harmful UV radiation while maximizing their access to PAR. Indeed, our light measurements showed that both UV light and PAR attenuate steeply in the stromatolites, making such optimization possible through phototaxis, a well known phenomenon in cyanobacteria [46]–[49]. Our measurements showed that the pronounced attenuation of the UV light in the stromatolites was due to the combined effects of scattering and absorption. However, more research is required to identify the responsible substances.

Despite the generally high UV levels in the ambient illumination, the photosynthetic activity in the stromatolites was high, comparable to other, low-altitude aquatic systems such as cyanobacterial mats or microalgal biofilms [50]. Generally, it was localized in a very thin (about 0.5 mm) subsurface layer with a highly concentrated biomass. This spatial arrangement is typical for benthic photosynthetic systems exposed to high light, and has been interpreted as a result of negative phototaxis towards PAR so as to prevent photoinhibition. However, our data suggest that in the Socompa stromatolites this is a result of negative phototaxis towards UV light. Indeed, when the excessive UV illumination was reduced while the PAR was kept at similar levels, the cyanobacterial population migrated up within a few days and formed a dense biofilm (about 0.2 mm thick) at the stromatolite surface. As a consequence, the net photosynthetic activity increased about 4.5-fold and 10-fold in terms of areal and volumetric rates, respectively, reaching values that are among the highest for benthic photosynthetic systems [50]. This demonstrates the magnitude of the detrimental effects of intense ambient UV radiation on photosynthetic activity, as well as highlights the importance of phototaxis as an adaptation that allows these effects to be minimized. Similar conclusions were reached based on observations of the same phenomenon in microbial mats from Solar Lake in Egypt [46].

### Bacterial Diversity Based on 16S Pyrotags

To put the microbial community in the Socompa stromatolites in a wider perspective with respect to its diversity and composition, we compared its Alpha-diversity metrics and performed a Beta-diversity analysis with other microbial ecosystems for which comparable data is available (see Supplement S2). This comparison revealed that, among the ecosystems whose habitat can be considered as extreme, the Socompa stromatolites can be generally classified as rich and very diverse. For example, the Shannon diversity index, the number of observed OTUs and equitability were higher than for all other extreme ecosystems except for the Guerrero Negro microbial mats. In contrast, these diversity metrics were considerably lower when compared with ecosystems from moderate habitats. With respect to the bacterial composition at the phylum level, the Socompa stromatolites are more similar to Guerrero Negro mats and Yellowstone stromatolites than to any other dataset used in the analysis (see Figure in Supplement S2).

An exciting finding of the sequence analysis is the presence in the Socompa stromatolites of highly abundant unclassified sequences with a very low identity to any known 16S sequence in the NCBI database. These sequences clustered into two groups that showed <80% identity to each other as well as to any other sequence in the whole dataset. Although there is no strict consensus between taxonomic levels and percentage identity, identities below 80% are usually considered as very distant lineages. When compared to the full NCBI database, the closest hits for the most abundant OTU in one of the groups were sequences from the phylum *Verrucomicrobia* (77% identity) and from the genus *Desulfotignum* (78% identity), two very distant lineages. For the other unclassified group, the closest hits included the mitochondrion of the diatom *Phaeodactylum tricornutum* (78% identity), and sequences from the family *Hyphomonadaceae* (76% identity) and genus *Sinorickettsia* (72% similarity), both assumed to be linked to an ancestor that gave rise to mitochondria through endosymbiosis [51]–[53]. Although the limited and short sequence information did not allow us to determine the nature of these two groups, it is clear that they represent novel and distant lineages.

To compare the Socompa stromatolites with other modern stromatolites, such as those from Shark Bay, Obsidian Pool, Cuatro Ciénegas, Highborne Cay and Ruidera Pools [41], [43], [44], [54]–[57], we analyzed the relative abundances of their most abundant taxonomic groups. We found that with respect to the bacterial phyla such as *Acidobacteria*, *Actinobacteria*, *Bacteroidetes*, *Chloroflexi, Firmicutes, Proteobacteria* and *Verrucomicrobia,* which are found in modern stromatolites with relative abundances in the range a few percent to tens of percent, the Socompa stromatolites are not that much different. However, there are a few notable differences, as discussed below.

*Deinococcus-Thermus* appears to be highly abundant in the Socompa stromatolites (7% of sequences). These aerobic heterotrophic bacteria are known for their UV resistance, associated with their photo-protective pigment deinoxanthin [58], [59]. Unfortunately, because we were not able to analyse the surficial white-pinkish crust by HPLC, we could not identify this pigment in the stromatolites. The high abundance of *Deinococcus* was also found in the Obsidian Pool stromatolites [41], which form at an altitude of 2400 m, whereas this phylum is generally minor in the low-altitude stromatolites. We hypothesize that *Deinococcus* proliferation is linked to the presence of high UV irradiance: their UV resistance gives them the selective advantage to outcompete other microorganisms in a UV-intensive, aerobic and organic-rich environment (e.g., at the stromatolite surface), and their special pigmentation, which is linked to this resistance, provides a shelter against UV light for the rest of the community.

Other comparatively over-represented groups in the Socompa stromatolites include *Deltaproteobacteria* (mostly *Desulfobacterales*; 12%) and *Spirochaetes* (8%). *Desulfobacterales* are sulphate reducing bacteria [60], whereas *Spirochaetes* are motile, anaerobic, facultative anaerobic, or microaerophilic bacteria with heterotrophic or chemo-organotrophic metabolism and with high tolerance towards sulfide [61]. The reason for their unusually high abundance could be related to the sampling of DNA, which included a large volume with active SR and high sulfide concentrations. In the context of the hypothesized intense episodic growth of diatoms at the stromatolite surface (see above), one can speculate that the motility of *Spirochaetes* could be an additional advantage that allows them to outcompete other bacteria with similar metabolism.

The Socompa stromatolites contained a very high abundance of *Alphaproteobacterial* sequences from the family *Rhodobacteraceae*, which are representative of photosynthetic, Bchl *a* containing purple non-sulfur bacteria. Although these bacteria are highly abundant also in other modern stromatolites, the peculiarity of the Socompa stromatolites lies in their spatial distribution. Assuming that the Bchl *a* pigment detected by HPLC and hyper-spectral imaging belonged to this group, our results suggest that they form distinct layers at depths ranging from several millimetres to centimetres. However, as shown by our light measurements, near infrared light, which can be utilized by these bacteria, is very likely extremely low at such depths. A similar peculiarity pertains to the Bchl *c* containing *Chloroflexi*, which are also found in distinct layers at depths where hardly any light penetrates. However, this group, although bearing photo-pigments, may not necessarily be using them for phototrophy. Indeed, Bchl *c* containing *Chloroflexi* have been found highly abundant across several centimetres in a microbial mat, colonizing polysaccharide sheaths produced by *Microcoleus* in oxic parts and pervading the polymeric matrix of the mat in deeper, anoxic parts of the mat [62]. Further experiments are required to elucidate the spatial distribution of these groups.

Other notable differences in the Socompa stromatolites included a very low abundance of *Planctomycetes* (0.06%) and an absence of green sulfur bacteria (*Chlorobi*). These groups are usually found in the range of a few percent in other modern stromatolites [44]. Also, *Cyanobacteria* appeared to be under-represented in the Socompa stromatolites, with only 3% of sequences in comparison to 10–70% in other stromatolites. This could be, however, again due to the fact that the volume with abundant cyanobacteria comprised only about 1–2% of the total volume sampled for DNA.

### Diversity of the *nif*H and *amo*A genes

Nitrogenase is an ancient enzyme that remained conserved during the transition from anoxic to oxic biosphere [63]. Since diazotrophs may have composed a key functional group in ecosystems on early Earth that developed in N-depleted waters, it is worth to examine their diversity in what is considered as their modern analogues, e.g. stromatolites. Our results suggest that the community with the diazotrophic potential in the Socompa stromatolites is rather diverse, comprising *Delta*-, *Gamma-* and *Betaproteobacteria*, *Cyanobacteria* (close relatives of *M. chthonoplastes*), and rather distant relatives of *Spirochaetes* and *Chlorobiales.* Interestingly, more than half of the *nif*H sequences in the clone library clustered with those of SRB, suggesting that SRB are highly abundant members of the stromatolites community, in agreement with the 16S pyrotag analysis. Although SRB have been previously reported as members of the diazotrophic community in marine systems and hypersaline mats [64]–[67], our results suggest that they may play a key role in the Socompa stromatolites.

Nitrification, the microbial oxidation of ammonia to nitrite and nitrate, occurs in a wide variety of environments and plays a central role in the global nitrogen cycle. This process is presently known to be performed by two groups of *Bacteria* (*Beta*- and *Gammaproteobacteria*) and by diverse groups of *Archaea* [68], [69]. Our results suggest that the diversity of the nitrifying community in the Socompa stromatolites is rather low, restricted to two *Betaproteobacterial* groups (*Nitrosomonas* and *Nitrosospira*). Similar results were obtained for high altitude lakes on the Tibetan Plateau and other inland water systems [70].

Interestingly, most of the *nif*H and *amo*A sequences detected in the Socompa stromatolites show low similarity (<85%, some of them even 70–75%) with those of cultivated isolates, suggesting that the nitrogen cycle in the stromatolites involves largely unknown microorganisms. Furthermore, their similarity to sequences from other saline environments suggests that these environments select and promote evolutionarily novel groups of microorganisms that can tolerate a wide range of salinities, and provides further evidence that salinity is among the most significant factors shaping the microbial community structure [71].

### Conclusion

To the best of our knowledge, the volcanic lake Socompa is so far the highest site with documented actively forming stromatolites. The Socompa stromatolites are characterized by features that are typical for what is classified as a modern stromatolite: they are layered microbial communities forming flat, domical or columnar macroscopic structures at the sediment-water interface, and contain a high abundance of minerals that most likely form by *in situ* precipitation. They are rich, diverse and active ecosystems that thrive in an environment characterized by a multitude of extremes, including high UV radiation, alkalinity, concentrations of arsenic and dissolved salts, and reduced atmospheric O_2_ partial pressure. Therefore, they are an outstanding model system for studying microbe-microbe and microbe-mineral interactions, physiological adaptations and microbial evolution under environmental conditions that likely prevailed during a large part of the Earth's history. Furthermore, due to their genetic richness, diversity and novelty, they may harbour genomic and proteomic reserves that may be of interest for future biotechnological applications. There are other high-altitude lakes in the Argentinean and Chilean Puna. Although they harbour similarly rich and diverse microbial communities [72]–[76], the development of stromatolites was, until now, only found in Socompa. These lakes are geographically remote and ecologicaly isolated, however their balance, or even existence, is threatened by the mining prospects. We hope that this study will provide a stimulus and basis for the efforts to preserve these unique ecosystems.

## Materials and Methods

### Sampling

Stromatolite and water samples were collected in sterile plastic bags and flasks during a campaign in February 2011. Samples for scanning electron microscopy (SEM), lithogeochemistry and water chemistry analyses were stored in the dark at 4°C and processed within 1–2 weeks. Samples for optical microscopy were fixed on site with 4% formaldehyde and analyzed within a few days. Samples for DNA and pigment extraction were frozen in liquid nitrogen, stored in the dark, and processed within a week. Unless stated otherwise, samples for microsensor measurements were kept in lake water at ambient temperature and light conditions. Permission for sample collection was granted by the Ministerio de Ambiente y Desarrollo Sustentable, Salta, Argentina (number 000388; 17–09–2010).

### Water Chemistry Analysis

Chemical analysis of water samples was done at Estación Experimental Obispo Colombres, San Miguel de Tucumán, Argentina.

### Lithogeochemistry Analysis

Lithogeochemical analysis of the stromatolite solid phase was done by inductively coupled plasma atomic emission spectroscopy (ICP-AES) and mass spectrometry (ICP-MS) at ALS Minerals labs in Argentina and Canada.

### SEM Analysis

Samples were fixed over night at 4°C in a Karnovsky fixative comprising formaldehyde (8% v/v), glutar-aldehyde (16% v/v) and phosphate buffer (pH 7). The fixed samples were washed three times with phosphate buffer and CaCl_2_ for 10 min, and fixed with osmium tetroxide (2% v/v) over night. Afterwards, the samples were washed twice with ethanol (30% v/v) for 10 min, dried at a critical point, and sputtered with gold. Specimens were observed under vacuum using a Zeiss Supra 55VP (Carl Zeiss NTS GmbH, Germany) scanning electron microscope.

### Microsensors Measurements

Scalar irradiance, E_s_, in the stromatolite was measured with a fiber-optic microprobe [77] connected to a spectrometer (USB 4000; Ocean Optics). The integrating sphere diameter was 200 µm. The direct measurement of E_s_ in the UV region was not possible due to the high light attenuation by the fiber-optics used. Therefore, it was estimated based on E_s_ measured at wavelengths 676 nm and 750 nm as described in Supplement S1. Oxygen, hydrogen sulfide and pH were measured with microsensors prepared as previously described [78]–[80]. The tip diameters were 30 µm (O_2_), 100 µm (H_2_S) and 50 µm (pH). Routine measurements of water temperature and conductivity were done with a digital TDS meter (AD32, ADWA Waterproof), of the intensity of photosynthetically active radiation (PAR, 400–700 nm) with a calibrated light logger (Odyssey, Dataflow Systems), and of the intensity of UV-B (280–320 nm) with a UV-B radiometer (9811-series, Cole-Parmer).

All microsensor measurements were conducted and analyzed as previously described [81]–[83]. The sample was placed in a container and covered with ∼1 cm of the lake water. A gentle air stream from a pipette was used to maintain the overlying water in equilibrium with the atmosphere and at continuous movement (velocity ∼1 cm s^−1^), the latter to ensure a stable diffusive boundary layer.

To characterize the microbial activity and physico-chemical microenvironments in the stromatolite under close to natural conditions, the measurements were conducted in Tolar Grande, a nearby (∼85 km) settlement characterized by similar altitude (3500 masl) and general environmental conditions as the Socompa lake. To study the effects of UV-B radiation on the cyanobacterial migration and on the photosynthetic and respiration activity in the stromatolite, additional microsensor measurements were conducted under controlled laboratory conditions in San Miguel de Tucumán (460 masl). In the latter measurements, artificial UV-B radiation was provided with a UV-B lamp (9815-series, Cole-Parmer).

Oxygen concentration in air-saturated lake water, required for the calibration of the O_2_ sensor**,** was calculated as PF×O_2_ solubility, where PF is the pressure factor that accounts for the decrease in ambient air-pressure with altitude (PF = 0.66 for 3500 masl; PF = 0.95 for 460 masl; www.altitude.org/air_pressure.php). The temperature- and salinity-corrected O_2_ solubility under normal air-pressure (at sea level) was calculated according to Ref. [84], where the salinity of the lake water was estimated based on its conductivity assuming similar salt composition as in seawater. Oxygen fluxes and volumetric rates of net photosynthesis were calculated based on Fick's law of diffusion, using the gradient (J = –D_eff_ (dc/dz)) and curvature (net PS = –D_eff_ (d^2^c/dz^2^)) of the measured steady state vertical profiles, where c denotes O_2_ concentration and z is depth [82]. In this calculation, the effective diffusion coefficient of O_2_ in the stromatolite was estimated as D_eff_ = φD_O2_, where φ is the porosity and D_O2_ is the temperature- and salinity-corrected molecular diffusion coefficient of O_2_ in water [85].

### Pigment Analyses

High spatial resolution 2D maps of pigments in the stromatolite were obtained by hyperspectral imaging [86]. Vertical pigment profiles were additionally obtained by reversed-phase high performance liquid chromatography (HPLC) and spectrophotometry. For these analyses, samples were sectioned along visible layers, stored in the dark in liquid nitrogen, and processed within a week in the laboratory in S. M. de Tucumán. Phycocyanin was extracted by incubating freeze-dried samples for 2 h at 37°C in a phosphate buffer (65 mM, pH 8.2) with added lysozyme (15 mg ml^−1^). Subsequently, the lysate was centrifuged at 3000 g for 20 min at 4°C, the supernatant's absorbance was measured in a spectrophotometer (Beckmann, DU640), and the pigment concentration was calculated previously described [87]. Chlorophylls and carotenoid pigments were extracted by overnight incubation of freeze-dried samples in 100% methanol at −20°C. Subsequently, the extracts were centrifuged at 8000 g for 10 min at 4°C, filtrated through 0.2 µm pore diameter syringe filters, and measured by HPLC [88]. The HPLC system consisted of two pumps (Waters, model 510), a syringe loading injector (Rheodyne 7125) fitted with 200 µl loop (Rheodyne 7025), and a photodiode array detector (Waters 996) coupled to a computer equipped with the Empower 2007 Chromatography Manager software (Waters-Millipore). The column used was Kinetex C18 (2.6 µm silica particles) protected by an Ultra In-Line Krudkatcher filter (Phenomenex). Pigments of interest were identified based on their absorption spectra and retention times [88], [89], and quantified as m = FA(e_m_d) ^−1^, where m is the pigment mass, F is the rate of solvent flow through the column (0.5 ml min^−1^), A is the time-integrated area of the elution peak, e_m_ is the extinction coefficient, and d is the detection length of the diode array detector (1 cm) [62]. The following extinction coefficients (in L g^−1^ cm^−1^) at the wavelength of maximal absorption were used: 79.95 for Chl *a* [90], 60 for BChl *a* [91], and 86 for BChl *c* [92]. In hyperspectral images, relative Chl *a* concentrations were estimated as the depth of the valley in the log-transformed reflectance at the wavelength of maximal *in vivo* Chl *a* absorption (676 nm), i.e., Chl *a* = log(R_750_/R_676_) [93].

### DNA Extraction

A small core (2.5 cm diameter; 5 cm deep) taken from the top of the stromatolite sample stored at −20°C was lyophilized and homogenized. The powder (0.5 g) was washed three times by adding 5 ml of 10% w/v NaCl, incubating at 40°C for 15 min to dissolve EPS (extracellular polymeric substances) in water [67], and centrifuging at 8200 g for 15 min. Extraction of the genomic DNA from the pellet was done as previously described [67], with the following modifications. First, the pellet was resuspended in 5 ml of lysis buffer (Tris-HCl, pH 8, 100 mM; NaCl 1.5 M; EDTA, pH 8, 100 mM; Na_3_PO4, pH 8, 100 mM; CTAB 1%). The cells were then frozen at −120°C and thawed in a water bath at 65°C, which was repeated three times. The mixture was subsequently incubated with lysozyme (1 mg ml^−1^) for 30 min at 37°C, followed by the addition of Proteinase K (0.1 mg ml^−1^) and 3% (w/v) final SDS (sodium dodecyl sulfate). After overnight incubation at 60°C, the supernatant was collected after 10 min of centrifugation at 10,000 g. Organic extractions were made first with 1 volume of phenol:chloroform: isoamyl alcohol (25∶24:1) and then with 1 volume of chloroform:isoamyl alcohol (24∶1). DNA (aqueous phase) was precipitated with cold isopropanol (0.7 vol.), incubated for 2 hours at −20°C, and then washed three times with cold 70% v/v ethanol, collecting the DNA between each wash by centrifugation for 15 min at 15000 g and 4°C. Finally, the DNA was dried in sterile air and resuspended in DNAse-free Milli-Q water.

### PCR Amplification and 454 Pyrosequencing

The V4 hyper-variable region of the Bacterial 16S rRNA gene was amplified using the universal primers suggested by the Ribosomal Database Project (RDP; http://pyro.cme.msu.edu/pyro/help.jsp). The primers contained the Roche 454 sequencing A and B adaptors and a 10 nucleotide “multiple identifier” (MID). PCR amplification was done on a FastStart High Fidelity PCR system (Roche Applied Science, Mannheim, Germany) following the manufacturer's instructions. Five independent PCRs were performed to reduce bias. Two negative controls with no template were also performed. The PCR conditions were 95°C for 5 min, followed by 30 cycles of 95°C for 45 s, 57°C for 45 s and 72°C for 60 s, and a final elongation step at 72°C for 4 min. The five reactions were pooled, purified and sequenced on a Genome Sequencer FLX (Roche Applied Science) at the INDEAR genome sequencing facility (Argentina) following the amplicon sequencing protocol provided by the manufacturer. A total of 113,255 filtered sequences with an average length of 250 bp were obtained. Filter parameters were set to reject reads that had mean quality score <25, maximum homopolymer run >6, number of primer mismatches >0, and read length <200 bp or >1000 bp. The sequences were deposited as FASTAQ in the NCBI Sequence Read Archive (SRA) under the accession number SRP007748.

### Taxonomy, Alpha and Beta Diversity Analyses

Taxonomic analysis of the 16S pyrotags was performed using the QIIME software v1.5.0 [94]. Sequences were aligned with the Pynast module included in QIIME using Silva database release 108 non-redundant template for QIIME (www.arb-silva.de/no_cache/download/archive/qiime/). Gap-only sites of the resulting alignment were eliminated. Classification was performed against the Greengenes database using the RDP classifier included in QIIME (bootstrap confidence of 50%).

Additionally, alpha and beta diversity metrics were calculated from the Socompa stromatolite sequences and compared to those obtained for samples collected at both extreme and moderate habitats (see Table in Supplement S2). In this analysis, sequences were clustered into operational taxonomic units (OTUs) with UCLUST at 0.97, 0.90 and 0.80 similarity. To enable comparison between all datasets, 1557 (i.e., the lowest number of sequences in the compared datasets) sequences were subsampled from each OTU table in 10 replicates and the metrics were averaged. Finally, a UPGMA tree was built in QIIME based on Bray Curtis distances between samples, which were calculated using BiodiversityR package based on the relative abundances at phylum level obtained in QIIME.

### *nif*H and *amo*A gene Libraries

The *nif*H gene was amplified using universal primers Z1 and Z2 [95]. The PCR amplification was done as previously described [96], with minor modifications: the thermal cycle consisted of 30 cycles of 94°C for 1 min, followed by 55°C for 1 min and 72°C for 2 min.

PCR amplification of the *amo*A gene was done using three sets of primers specific for each known group of ammonium oxidisers. The archaeal sequences were amplified according to Ref. [69], using Arch-*amo*AF and Arch-*amo*AR as forward and reverse primers. The γ-proteobacterial *amo*A gene was amplified with amoA49f-amoA627r primers as previously described [97]. The β-proteobacterial *amo*A gene was amplified with the primers amoA-1F-amoA-2R [98], using annealing temperature of 54°C.

Phylogenetic analyses were performed separately on 321 bp of the *nif*H gene and 440 bp of the *amo*A gene using MEGA software v4.0 [99]. Sequences were aligned using the ClustalW module included in MEGA (default settings). To assess the *amo*A and *nif*H gene libraries for diversity and phylotype coverage, rarefaction analysis and CHAO1 index were calculated using the DOTUR software [100]. OTUs were defined at 0.95 similarities, which corresponds roughly to the level of microbial species in conserved protein-encoding sequences [101]. The sequences were deposited in GenBank under accession numbers JX134223 to JX134261 (*nif*H) and JX154589 to JX154609 (*amo*A).

## Supporting Information

Supplement S1 **Estimation of the UV light attenuation in the stromatolite.**
(PDF)

Supplement S2 **Alpha-diversity metrics and Beta-diversity analysis of microbial communities from different habitats.** The compared samples/habitats include the Socompa stromatolite (SRA accession number SRP007748, benthic; this study), the Red Sea water column (SRX020658, pelagic; [102]), the Jan Mayen hydrothermal vent field (SRP004929, benthic; [103]), the Dead Sea (ERA116549, diverse; [104]), El Zacatón sinkhole, Mexico (SRX003633, pelagic; [105]), Atacama hyper-arid soils (SRA030747; [106]), sub-tropical marine biofilms (SRA029303, benthic; unpublished), Yellowstone stromatolites (http://inside.mines.edu/~jspear/resources.html, benthic; [45]), Highborne Cay thrombolites, Bahamas (SRX030166, benthic; [107]), biofilms from a high-altitude lake Diamante, Argentina (MGRAST 4493670.3, benthic; Rascovan, unpublished data), and the Guerrero Negro microbial mat, Mexico (GenBank accession numbers DQ329539 to DQ331020 and DQ397339 to DQ397511, benthic; [62]). The analyses were done on subsampled datasets containing 1557 sequences (corresponding to the number of sequences in the smallest dataset). Table shows selected alpha-diversity metrics calculated at different similarity levels. E and N denote habitats whose environmental settings can be considered as extreme and moderate, respectively. Figure shows the similarities among the compared communities, as derived from the Bray Curtis metric calculated at phylum level. The number of OTUs at 97% identity and the Shannon biodiversity indices are also shown (taken from the table).
(PDF)
