# Supplementary material for: The Discovery of Stromatolites Developing at 3570 m above Sea Level in a High-Altitude Volcanic Lake Socompa, Argentinean Andes
Source: PLoS One. 2013 Jan 7;8(1):e53497. doi: 10.1371/journal.pone.0053497 (PMC3538587; doi:10.1371/journal.pone.0053497)
Supplement: Supplement S1 — Estimation of the UV light attenuation in the stromatolite. (PDF) [file pone.0053497.s001.pdf]

Supplement 1: Estimation of the UV light attenuation in the stromatolite

The attenuation of UV light in the stromatolite was estimated using the followig steps. First, we experimentally verified that the stromatolite's absorbance at a specific wavelength λ can be expressed as a **sum** of two components, light absorption and light scattering, i.e.,

$$A_\lambda = e_\lambda c_p + S_\lambda, \tag{1}$$

where e_λ (in $\mathrm{ml}\,\mu\mathrm{g}^{-1}\,\mathrm{cm}^{-1}$) and c_p (in $\mu\mathrm{g}\,\mathrm{ml}^{-1}$) are, respectively, the extinction coefficient and concentration of the absorbing substance (e.g., a pigment), and S_λ (in cm^{-1}) is the absorbance due to scattering (e.g., by particles). Note that without loss of generality it is sufficient to consider only one pigment as a contributor to light absorption.

To verify Eq. (1), pigments in a stromatolite sample (top 1 mm) were first extracted with methanol to physically separate the absorbing and scattering components. The pigment-free stromatolite matrix was then diluted with water, and its transmission, T_λ, was measured in a quarz cuvette ($\Delta z = 1\,\mathrm{cm}$) using a spectrometer sensitive at wavelengths 250–800 nm (Beckmann, DU640). The absorbance due to scattering was then calculated as $S_\lambda = (\Delta z)^{-1}\log(1/T_\lambda)$. This was done at different dilutions of the stromatolite matrix, and revealed that S_λ slightly increased at shorter wavelengths (Figure S1a) and scaled with the concentration of the solids in the turbid sample as

$$S_\lambda(c_s) = S_{\max} c_s/(K_s + c_s), \tag{2}$$

where both $S_{\max}$ and K_s depended on wavelength (see below). Subsequently, the extracted pigments were added at different amounts to the turbid samples, the transmission was measured, and the absorbance calculated as $A_\lambda = (\Delta z)^{-1}\log(1/T_\lambda)$ (Figure S1b). Because the concentrations of solids in these samples were known, their contribution to light scattering could be estimated based on Eq. (2) (dashed lines in Figure S1b), making it possible to quantify the contribution of the pigments to the measured absorbance. In the interval of measured wavelengths, **this contribution scaled linearly with the pigment concentrations (Figure S1c), demonstrating empirically the validity of Eq. (1).**

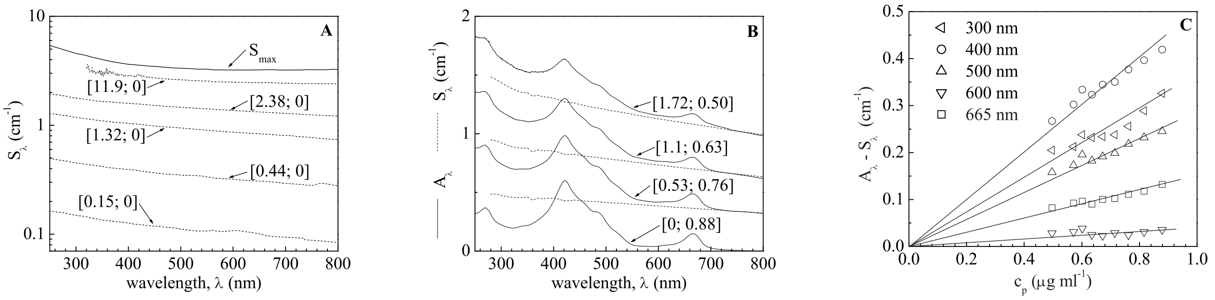

Figure S1: Absorbance measurements in turbid stromatolite samples diluted in water. The values in brackets, $[c_s; c_p]$, specify the concentrations of solids (c_s, in $\mathrm{mg\,ml^{-1}}$) and pigments (c_p, in $\mu\mathrm{g\,ml^{-1}}$) in the sample. Dashed lines in panel B show the contribution due to scattering, as derived from the measurements in pigment-free samples shown in panel A. Absorbance due to pigments was calculated by subtracting this contribution, and is shown at different wavelengths as a function of the pigment concentration in panel C.

In the second step, we assumed that the wavelength-dependent scalar irradiance, $E_{s,\lambda}$, decreases exponentially with depth z in the stromatolite according to

$$E_{s,\lambda}(z)/E_{s,\lambda}(0) = 10^{-A_\lambda z}. \tag{3}$$

Because the concentration of pigments and solids could not be measured in the very same point of an intact stromatolite sample where the microprobe was used to measure the scalar irradiance, $E_{s,\lambda}$ could not be pedicted simply by calculating A_λ based on Eq. (1) and substituting it into Eq. (3). However, by taking advantage of the additive form of A_λ, which is the reason why we conducted the validation experiments described above, scalar irradiance at a specific wavelength λ can be estimated based on the measurements of scalar irradiances

at two other wavelengths, λ_1 and λ_2, as described in the following. Specifically, based on Eq. (1), Eq. (3) can be rewritten as

$$E_{s,\lambda}(z)/E_{s,\lambda}(0) = 10^{-e_\lambda c_p z} \times 10^{-S_\lambda z} = \left(10^{-e_{\lambda i} c_p z}\right)^{e_\lambda/e_{\lambda i}} \times \left(10^{-S_{\lambda j} z}\right)^{S_\lambda/S_{\lambda j}}, \quad (4)$$

where the choice of i and j can be arbitrary (1 or 2). The individual terms in this equation are generally unknown, but can be determined experimentally if one of the wavelengths is chosen such that $e_{\lambda 1} = 0$ and $S_{\lambda 1} > 0$ whereas the other is chosen such that $e_{\lambda 2} > 0$ and $S_{\lambda 2} > 0$. For example, if pigments such as bacteriochlorophylls, which absorb in the near infrared region, are not significant in the sample, whereas pigments such as chlorophyll *a* are, a possible choice would be $\lambda_1 = 750$ nm and $\lambda_2 = 676$ nm (maximal *in vivo* absorption of Chl *a*). Applying Eq. (4) and assuming $e_{\lambda 1} = 0$, the decrease in scalar irradiance at these wavelengths is given by

$$\begin{aligned} E_{s,\lambda 1}(z)/E_{s,\lambda 1}(0) &= \left(10^{-S_{\lambda 2} z}\right)^{S_{\lambda 1}/S_{\lambda 2}} \\ E_{s,\lambda 2}(z)/E_{s,\lambda 2}(0) &= 10^{-e_{\lambda 2} c_p z} \times 10^{-S_{\lambda 2} z}. \end{aligned} \quad (5)$$

By a simple rearrangement of Eqs. (4–5), and for the above choice of λ's, one can write

$$\frac{E_{s,\lambda}(z)}{E_{s,\lambda}(0)} = \left(\frac{E_{s,676}(z)}{E_{s,676}(0)}\right)_{\text{meas}}^{r_e} \times \left(\frac{E_{s,750}(z)}{E_{s,750}(0)}\right)_{\text{meas}}^{r_S}, \quad (6)$$

where the exponents are calculated as

$$\begin{aligned} r_e &= \tfrac{e_\lambda}{e_{676}} \\ r_S &= \tfrac{S_\lambda}{S_{750}} - \tfrac{e_\lambda}{e_{676}} \tfrac{S_{676}}{S_{750}}. \end{aligned} \quad (7)$$

Thus, **by measuring the scalar irradiance profile at wavelengths 676 nm and 750 nm (terms inside the parentheses in Eq. (6)), and by measuring the ratios between the extinction and scattering coefficients e_λ/e_{676}, S_λ/S_{750} and S_{676}/S_{750}, one can use Eqs. (6–7) to estimate the scalar irradiance profile at an arbitrary wavelength λ.**

The concentration of solids in an intact Socompa stromatolite (about 0.55 $\mathrm{g\,ml^{-1}}$) were much greater than the value of K_s determined from the data presented in Figure S1 (0.0035–0.0038 $\mathrm{g\,ml^{-1}}$). This implied that the absorbance strength due to scattering was $S_\lambda \approx S_{\max}$ (dashed line in Figure S1a). Thus, the ratios S_λ/S_{750} ranged from 1.1 to 1.5 in the UV region (280–400 nm) and S_{676}/S_{750} was about 1 (Figure S2, symbols). Furthermore, the extinction coefficients of the methanol-extracted pigments were negligible at 750 nm and the ratio e_λ/e_{676} ranged from 1 to 2 in the UV region (Figure S2, solid line). This range of values, in combination with the measured scalar irradiances at 676 nm and 750 nm (see Figure 3a in the manuscript) and Eqs. (6–7), was used to calculate the scalar irradiance in the stromatolite in the UV region.

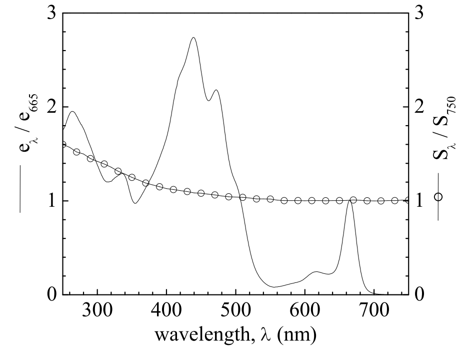

Figure S2: Absorbance of methanol-extractable compounds in the Socompa stromatolite (solid line), and the strength of light scattering by the particles in the stromatolite (symbols). The spectra were normalized at wavelengths 665 nm and 750 nm, respectively. Note that the wavelengths of maximal Chl *a* absorption differ when measured *in vivo* (676 nm) an in a methanol extract (665 nm). For this reason, the absorbance of the extract was normalized at 665 nm, while the calculations of the scalar irradiance profile were based on the scalar irradiance measurements at 676 nm. Also note that the absorption of light in the UV region may be not only due to pigments, but also due to other components in the stromatolite, such as DNA, proteins, phenolic compounds. However, this distinction is not important in the context of light penetration measurements in this study.
