# Supplementary material for: The Discovery of Stromatolites Developing at 3570 m above Sea Level in a High-Altitude Volcanic Lake Socompa, Argentinean Andes
Source: PLoS One. 2013 Jan 7;8(1):e53497. doi: 10.1371/journal.pone.0053497 (PMC3538587; doi:10.1371/journal.pone.0053497)
Supplement: Supplement S2 — Alpha-diversity metrics and Beta-diversity analysis of microbial communities from different habitats. The compared samples/habitats include the Socompa stromatolite (SRA accession number SRP007748, benthic; this study), the Red Sea water column (SRX020658, pelagic; [102]), the Jan Mayen hydrothermal vent field (SRP004929, benthic; [103]), the Dead Sea (ERA116549, diverse; [104]), El Zacatón sinkhole, Mexico (SRX003633, pelagic; [105]), Atacama hyper-arid soils (SRA030747; [106]), sub-tropical marine biofilms (SRA029303, benthic; unpublished), Yellowstone stromatolites (http://inside.mines.edu/~jspear/resources.html, benthic; [45]), Highborne Cay thrombolites, Bahamas (SRX030166, benthic; [107]), biofilms from a high-altitude lake Diamante, Argentina (MGRAST 4493670.3, benthic; Rascovan, unpublished data), and the Guerrero Negro microbial mat, Mexico (GenBank accession numbers DQ329539 to DQ331020 and DQ397339 to DQ397511, benthic; [62]). The analyses were done on subsampled datasets containing 1557 sequences (corresponding to the number of sequences in the smallest dataset). Table shows selected alpha-diversity metrics calculated at different similarity levels. E and N denote habitats whose environmental settings can be considered as extreme and moderate, respectively. Figure shows the similarities among the compared communities, as derived from the Bray Curtis metric calculated at phylum level. The number of OTUs at 97% identity and the Shannon biodiversity indices are also shown (taken from the table). (PDF) [file pone.0053497.s002.pdf]

Supplement 2: Alpha-diversity metrics and Beta-diversity analysis of microbial communities from different habitats. The compared samples/habitats include the Socompa stromatolite (SRA accession number SRP007748, benthic; this study), the Red Sea water column (SRX020658, pelagic; [102]), the Jan Mayen hydrothermal vent field (SRP004929, benthic; [103]), the Dead Sea (ERA116549, diverse; [104]), El Zacatón sinkhole, Mexico (SRX003633, pelagic; [105]), Atacama hyper-arid soils (SRA030747; [106]), sub-tropical marine biofilms (SRA029303, benthic; unpublished), Yellowstone stromatolites (http://inside.mines.edu/~jspear/resources.html, benthic; [45]), Highborne Cay thrombolites, Bahamas (SRX030166, benthic; [107]), biofilms from a high-altitude lake Diamante, Argentina (MGRAST 4493670.3, benthic; Rascovan, unpublished data), and the Guerrero Negro microbial mat, Mexico (GenBank accession numbers DQ329539 to DQ331020 and DQ397339 to DQ397511, benthic; [62]). The analyses were done on subsampled datasets containing 1557 sequences (corresponding to the number of sequences in the smallest dataset). Table shows selected alpha-diversity metrics calculated at different similarity levels. E and N denote habitats whose environmental settings can be considered as extreme and non-extreme, respectively. Figure shows the similarities among the compared communities, as derived from the Bray Curtis metric calculated at phylum level. The number of OTUs at 97% identity and the Shannon biodiversity indices are also shown (taken from the table).

Supplement 2: Alpha-diversity metrics

		CHAO 1 *	Dominance **	Equitability **	Observed OTU *	Shannon Biodiversity Index **	Simpson **
Yellowstone Stromatolite exposed to air (E)	OTU at 97%	139	0.152	0.592	88	3.83	0.848
		170	0.145	0.599	85	3.84	0.855
	OTU at 90%	57	0.220	0.574	41	3.06	0.780
		52	0.176	0.619	37	3.23	0.824
	OTU at 80%	57	0.220	0.574	41	3.06	0.780
		52	0.176	0.619	37	3.23	0.824
Yellowstone Stromatolite Submerged (E)	OTU at 97%	169	0.113	0.693	129	4.85	0.887
		179	0.384	0.459	114	3.13	0.616
	OTU at 90%	78	0.152	0.653	70	4.01	0.848
		70	0.383	0.454	50	2.56	0.617
	OTU at 80%	78	0.152	0.653	70	4.01	0.848
		70	0.383	0.454	50	2.56	0.617
Dead Sea White Biofilm (E)	OTU at 97%	203	0.230	0.508	113	3.47	0.770
		206	0.211	0.537	114	3.67	0.789
	OTU at 90%	79	0.289	0.485	46	2.68	0.711
		70	0.270	0.516	50	2.90	0.730
	OTU at 80%	44	0.306	0.492	29	2.38	0.694
		46	0.277	0.525	34	2.66	0.723
Dead Sea Spring Sediments (E)	OTU at 97%	186	0.558	0.335	121	2.32	0.442
	OTU at 90%	99	0.673	0.266	72	1.64	0.327
	OTU at 80%	66	0.692	0.246	51	1.40	0.308
Dead Sea Green biofilm (E)	OTU at 97%	336	0.150	0.556	162	4.08	0.850
		341	0.157	0.545	157	3.98	0.843
	OTU at 90%	93	0.191	0.539	59	3.17	0.809
		107	0.190	0.535	65	3.21	0.810
	OTU at 80%	53	0.221	0.545	34	2.78	0.779
		55	0.203	0.560	38	2.92	0.797
Dead Sea Sediments (E)	OTU at 97%	246	0.052	0.761	188	5.75	0.948
	OTU at 90%	154	0.059	0.756	131	5.32	0.941
	OTU at 80%	119	0.141	0.664	85	4.25	0.859
Dead Sea Spring Water (E)	OTU at 97%	659	0.153	0.601	312	4.98	0.847
	OTU at 90%	418	0.159	0.560	222	4.36	0.841
	OTU at 80%	200	0.247	0.523	140	3.73	0.753
Atacama Soil at 2728 masl (E)	OTU at 97%	321	0.132	0.615	162	4.51	0.868
	OTU at 90%	118	0.151	0.592	83	3.77	0.849
	OTU at 80%	37	0.324	0.508	23	2.30	0.676
Atacama Soil at 4518 masl (E)	OTU at 97%	504	0.021	0.813	282	6.62	0.979
	OTU at 90%	141	0.057	0.765	109	5.18	0.943
	OTU at 80%	31	0.155	0.691	29	3.36	0.845
Marine Vent Hydrothermal (E)	OTU at 97%	523	0.092	0.675	235	5.32	0.908
	OTU at 90%	247	0.146	0.596	139	4.24	0.854
	OTU at 80%	65	0.279	0.504	47	2.80	0.721
Diamante Lake Biofilm (E)	OTU at 97%	820	0.041	0.759	371	6.48	0.959
	OTU at 90%	412	0.066	0.704	208	5.42	0.934
	OTU at 80%	112	0.151	0.602	80	3.81	0.849
Socompa stromatolite (E)	OTU at 97%	799	0.022	0.804	371	6.87	0.978
	OTU at 90%	376	0.039	0.748	212	5.78	0.961
	OTU at 80%	137	0.079	0.676	100	4.50	0.921
Guerrero Negro Microbial Mat (E)	OTU at 97%	864	0.009	0.884	469	7.84	0.991
	OTU at 90%	420	0.020	0.832	293	6.82	0.980
	OTU at 80%	118	0.075	0.715	82	4.54	0.925
Highborne Cay Pink (N)	OTU at 97%	1167	0.010	0.886	547	8.06	0.990
	OTU at 90%	563	0.019	0.828	326	6.91	0.981
	OTU at 80%	201	0.093	0.667	126	4.66	0.907
Highborne Cay Button (N)	OTU at 97%	1242	0.008	0.896	596	8.26	0.992
	OTU at 90%	568	0.021	0.817	289	6.68	0.979
	OTU at 80%	154	0.096	0.674	120	4.65	0.904
Highborne Cay Black (N)	OTU at 97%	1350	0.009	0.886	616	8.21	0.991
	OTU at 90%	531	0.027	0.803	332	6.73	0.973
	OTU at 80%	179	0.097	0.662	113	4.51	0.903
Highborne Cay Beige (N)	OTU at 97%	1334	0.005	0.918	641	8.56	0.995
	OTU at 90%	555	0.016	0.842	334	7.06	0.984
	OTU at 80%	193	0.070	0.709	130	4.98	0.930
Red Sea (N)	OTU at 97%	1840	0.027	0.837	642	7.80	0.973
	OTU at 90%	775	0.040	0.786	370	6.70	0.960
	OTU at 80%	276	0.055	0.724	165	5.33	0.945
El Zacatón sinkhole (N)	OTU at 97%	3036	0.013	0.860	774	8.25	0.987
	OTU at 90%	1641	0.026	0.815	578	7.48	0.974
	OTU at 80%	572	0.043	0.746	297	6.13	0.957
Sub-tropical marine biofilms (N)	OTU at 97%	6326	0.005	0.927	1019	9.27	0.995
	OTU at 90%	1988	0.028	0.818	638	7.62	0.972
	OTU at 80%	334	0.047	0.750	204	5.75	0.953

* Values did not reach an asymptote in the rarefaction curve.

** Values reached an asymptote in the rarefaction curve.

Datasets were normalized to 1557 reads to be comparable.

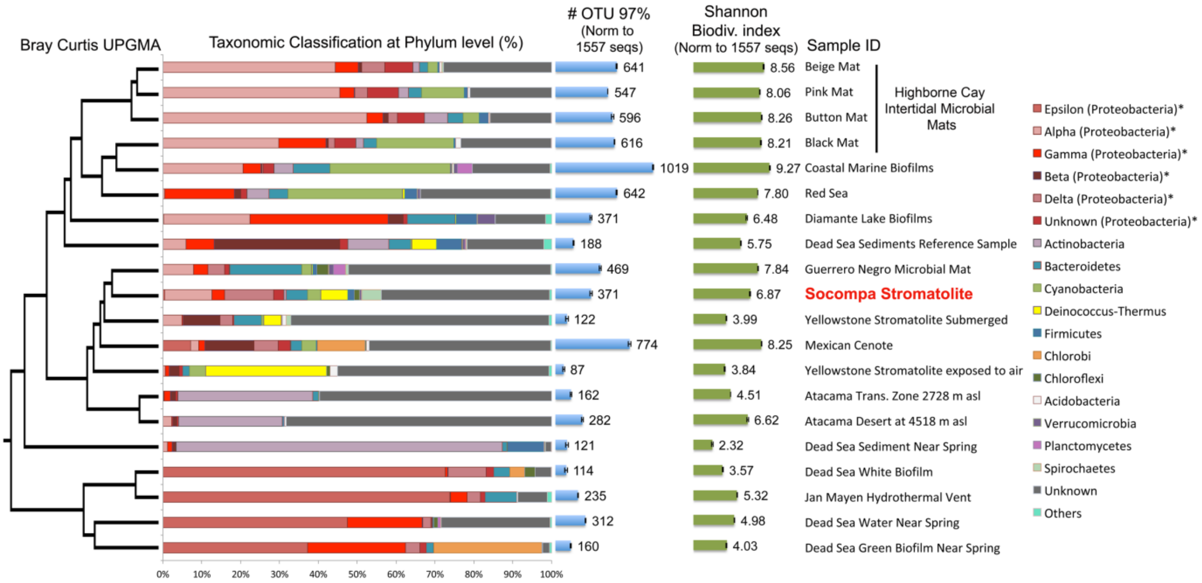
Bray Curtis UPGMA
Taxonomic Classification at Phylum level (%)
OTU 97% (Norm to 1557 seqs)
Shannon Biodiv. index (Norm to 1557 seqs)
Sample ID
641
8.56
Beige Mat
547
8.06
Pink Mat
596
8.26
Button Mat
616
8.21
Black Mat
Highborne Cay Intertidal Microbial Mats
1019
9.27
Coastal Marine Biofilms
642
7.80
Red Sea
371
6.48
Diamante Lake Biofilms
188
5.75
Dead Sea Sediments Reference Sample
469
7.84
Guerrero Negro Microbial Mat
371
6.87
Socompa Stromatolite
122
3.99
Yellowstone Stromatolite Submerged
774
8.25
Mexican Cenote
87
3.84
Yellowstone Stromatolite exposed to air
162
4.51
Atacama Trans. Zone 2728 m asl
282
6.62
Atacama Desert at 4518 m asl
121
2.32
Dead Sea Sediment Near Spring
114
3.57
Dead Sea White Biofilm
235
5.32
Jan Mayen Hydrothermal Vent
312
4.98
Dead Sea Water Near Spring
160
4.03
Dead Sea Green Biofilm Near Spring
0%
10%
20%
30%
40%
50%
60%
70%
80%
90%
100%
Epsilon (Proteobacteria)*
Alpha (Proteobacteria)*
Gamma (Proteobacteria)*
Beta (Proteobacteria)*
Delta (Proteobacteria)*
Unknown (Proteobacteria)*
Actinobacteria
Bacteroidetes
Cyanobacteria
Deinococcus-Thermus
Firmicutes
Chlorobi
Chloroflexi
Acidobacteria
Verrucomicrobia
Planctomycetes
Spirochaetes
Unknown
Others
